# Supplementary material for: Unpredicted Aberrant Splicing Products Identified in Postmortem Sudden Cardiac Death Samples
Source: Int J Mol Sci. 2022 Oct 20;23(20):12640. doi: 10.3390/ijms232012640 (PMC9604081; doi:10.3390/ijms232012640)
Supplement: Supplementary file 1 [file ijms-23-12640-s001.zip › ijms-1918956-supplementary.pdf]

Supplementary Materials

Table S1. Score of *in silico* predictions.

				SPLICEAI				MAXENTSCORE			
**Case**	**Gene**	**Variant**	**Existing Variation**	**DS_AG**	**DS_AL**	**DS_DG**	**DS_DL**	**MaxEntScan alt**	**MaxEntScan diff**	**MaxEntScan ref**	**Overall Prediction**
**#1**	*ANK2*	c.4248+9T>C	rs776783690	0.00	0.00	0.00	0.00	7.635	0.762	8.397	No effect
**#2**	*ANK2*	c.3224+7G>A	rs376534098	0.00	0.00	0.09	0.00				No effect
**#3**	*ANK2*	c.3379+4C>T	rs752152002	0.00	0.00	0.00	0.00				No effect
**#4**	*LAMA4*	c.3089+10A>G									No effect
**#5**	*MYBPC3*	c.1226+6T>C	rs397515892	0.01	0.02	0.02	0.03	7.470	2.607	10.077	No effect
**#6**	*MYPN*	c.2703+1G>T		0.00	0.00	0.00	0.99	2.262	8.504	10.766	High
**#7**	*RYR2*	c.13476+8_13476+11dup		0.00	0.01	0.00	0.06				No effect
**#8**	*SCN10A*	c.3228+9G>A	rs960885372	0.00	0.00	0.01	0.35				Low
**#9**	*SLMAP*	c.198+5T>C	rs759043349	0.00	0.00	0.00	0.01	10.193	1.200	8.993	No effect
**#10**	*TGFBR3*	c.568+1G>A	COSV53029765	0.00	0.00	0.03	0.92	0.702	8.182	8.884	High
**#11**	*TTN*	c.32245+5A>G	rs751566884	0.00	0.00	0.01	0.00	9.788	1.944	7.844	No effect
**#12**	*TTN*	c.46177+5G>A	rs753527304	0.00	0.00	0.00	0.10	3.971	4.196	8.167	No effect
**#13**	*MYH6*	c.3343-3 C>A	rs565446762	0.00	0.00	0.00	0.00	4.664	3.473	8.137	No effect
**#14**	*AKAP9*	c.9579-5C>G	rs1198599178	0.67	0.05	0.00	0.00	4.475	5.352	9.827	Moderate
**#15**	*MYH6*	c.20512del	rs1269980438	0.73	1.00	0.00	0.00				High
**#16**	*TMEM43*	c.1000+6T>C	rs773749788	0.00	0.00	0.17	0.00	.6029	0.829	6.858	No effect
**#17**	*DMD*	c.10921+5G>A	rs398123848, COSV58959263	0.00	0.00	0.00	0.18	4.687	5.552	10.239	No effect
**#18**	*FLNC*	c.5843-4C>T		0.00	0.00	0.00	0.00	7.651	1.082	8.733	No effect
**#19**	*TNNT2*	c.6904G>T	rs201753429	0.00	0.00	0.00	0.00	9.831	0.400	10.231	No effect
**#20**	*RYR2*	c.147576C>T	rs779122362	0.00	0.00	0.00	0.00	7.803	816	8.620	No effect
**#21**	*CACNA2D1*	c.659-2A>T	rs866568889, COSV62379849					2.710	8.367	11.077	High
**#22**	*TRDN*	c.132210T>A	rs764565579					4.572	6.711	11.283	No effect
**#23**	*TTN*	c.301796_301795insG									No effect

**#24**	*FLNC*	c.6998-5C>T	rs139030003	0.00	0.00	0.00	0.00	7.448	0.799	8.247	No effect
**#25**	*RYR2*	c.8831-9A>C	rs187977513	0.00	0.00	0.00	0.00	6.703	1.443	5.261	No effect
**#26**	*TTN*	c.31742-9T>A									No effect
**#27**	*TNNT2*	c.690-6G>A	rs113471285,COSV52664955	0.01	0.00	0.00	0.00	10.155	0.76	10.231	No effect
**#28**	*DMD*	c.9225-10T>C		0.00	0.12	0.00	0.00	8.421	0.874	9.295	No effect

Table S2. *In silico* predictors accuracy.

	Experimental Results			
	**Canonical Splice Variant**		**Deep Intron Variants**	
Prediction	+	-	+	-
+	*3*<br>*true positive*	*1*<br>*false positive*	*0*<br>*true positive*	*1*<br>*false positive*
-	*1*<br>*false negative*	*9*<br>*true negative*	*2*<br>*false negative*	*11*<br>*true negative*
**Prediction reliability parameters**	*Accuracy 85.7%*<br>*Sensitivity 75%*<br>*Specificity 90%*		*Accuracy 78.6%*<br>*Sensitivity 0%*<br>*Specificity 83.3%*	
	Accuracy 89.3%<br>*Sensitivity 50%*<br>*Specificity 90%*			

Table S3. Genes included in the custom NGS panel.

ABCC9, ACTC1, ACTN2, AKAP9, ANK2, BAG3, CACNA1C, CACNA2D1, CACNB2, CASQ2, CAV3, CRYAB, CSRP3, DES, DMD, DMPK, DSC2, DSG2, DSP, EMD, FBN1, FKTN, FLNA, FLNC, GLA, GPD1L, HCN4, JPH2, JUP, KCND3, KCNE1, KCNE2, KCNE3, KCNE5, KCNH2, KCNJ2, KCNJ5, KCNJ8, KCNQ1, LAMP2, LDB3, LMNA, MYBPC3, MYH6, MYH7, MYL2, MYL3, MYOZ2, MYPN, NEBL, NEXN, NOS1AP, PDLIM3, PKP2, PLN, PRKAG2, RANGRF, RBM20, RYR2, SCN1B, SCN2B, SCN3B, SCN4B, SCN5A, SCN10A, SGCD, SLMAP, SNTA1, TAZ, TCAP, TGFB3, TGFBR2, TMEM43, TMPO, TNNC1, TNNI3, TNNT2, TP63, TPM1, TRDN, TRIM63, TRPM4, TTN, TTR, VCL

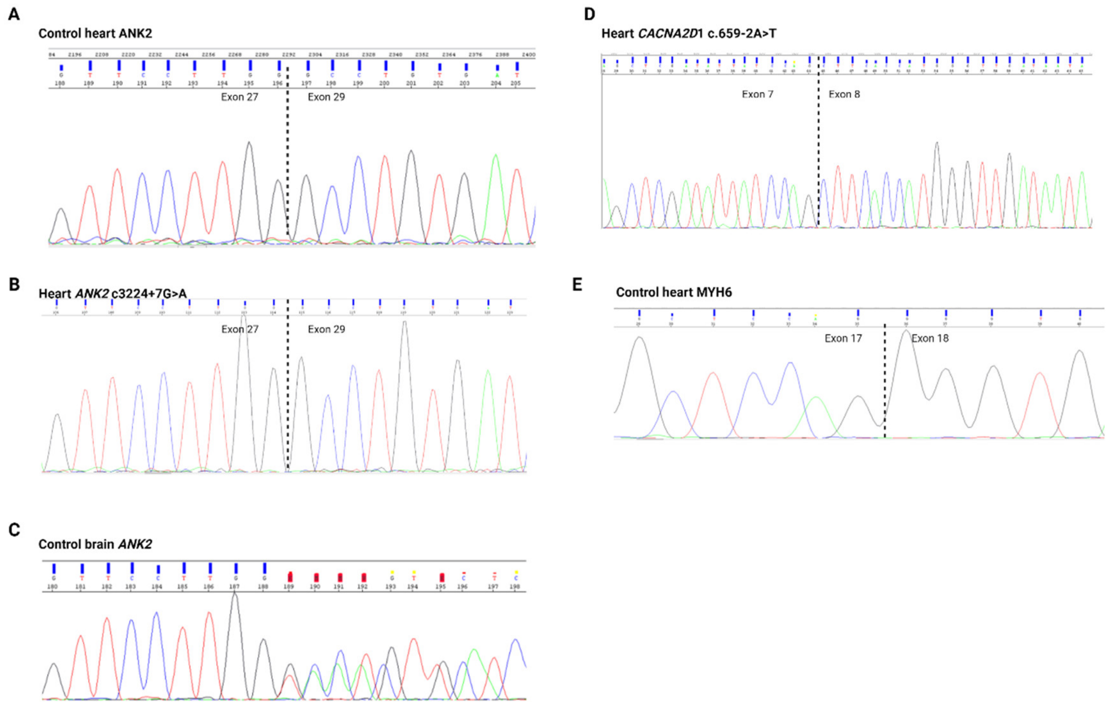

Figure S1. Genetic sequences. (**a**) *ANK2* exon 28 not expressed heart in control sample and (**b**) case sample. (**c**) *ANK2* exon 28 expressed in brain. (**d**) Normal splicing product for *CACNA2D1* c.6592A>T. (**e**) M*YH6* exon 18 expressed in control heart.
